# The predicting factors of chronic pain among nursing students: a national study in Iran

**DOI:** 10.1186/s40359-024-01803-9

**Published:** 2024-05-27

**Authors:** Maryam Shaygan, Banafsheh Tehranineshat, Saeed Hosseini Teshnizi, Agrin Mohammadi

**Affiliations:** 1 Community Based Psychiatric Care Research Center, School of Nursing and Midwifery, Shiraz University of Medical Sciences, Shiraz, Iran; 2 Department of Nursing, Faculty of Nursing and Midwifery, Hormozgan University of Medical Sciences, Bandar Abbas, Iran; 3 Department of Community Medicine, School of Medicine, Hormozgan University of Medical Sciences, Bandar Abbas, Iran; 4 Student Research Committee, School of Nursing and Midwifery, Shiraz University of Medical Sciences, Shiraz, Iran

**Keywords:** Nursing students, Chronic pain, Depression, Anxiety, Emotional intelligence, Academic performance, Social support

## Abstract

**Background:**

Nursing students are faced with a variety of challenges that demand effective cognitive and emotional resources. The physical and psychological well-being of the students plays a key part in the public health of the community. Despite the special lifestyle of nursing students, few studies have addressed chronic pain in this population. Accordingly, the present study aims to identify the predictors of chronic pain among nursing students.

**Methods:**

This cross-sectional study was conducted on 1,719 nursing students aged 18–42 years, between February and November 2019. Sampling was carried out in several stages. Data were collected using seven instruments, namely a demographics survey, the characteristics of chronic pain form, Spielberger State-Trait Anxiety Inventory (STAI), the Patient Health Questionnaire-9 (PHQ-9), the Bar-on Emotional Quotient Inventory, Academic Satisfaction Scale, and Procidano and Heller Social Support Scale. Descriptive statistics, multinomial logistic regression, and regression models were used to describe the characteristics of the pain and its predictive factors.

**Results:**

The average age of the participants was 22.4 ± 2.96 years. The results of univariate analysis showed that gender (*P* = 0.506), mother’s education (*P* = 0.056, *P* = 0.278, *P* = 0.278), father’s education (*P* = 0.817, *P* = 0.597, *P* = 0.41), place of residence (*P* = 0.215), depression (*P* = 0.501), grade point average (*P* = 0.488), academic satisfaction (*P* = 0.183) and chronic pain weren’t significantly correlated with chronic pain in nursing students. The results of the multiple logistic regression models showed that chronic pain was positively correlated with age, social support, state anxiety, and trait anxiety (OR = 1.07, 95% CI: 1.02–1.12; OR = 0.95, 95% CI: 0.93–0.97; OR = 1.03, 95% CI: 1.02–1.05; and OR = 1.97, 95% CI: 0.95–1.99; respectively).

**Conclusion:**

The prevalence of chronic pain was relatively high in these students. In addition, age, social support, and anxiety could be important factors in the development or persistence of chronic pain in nursing students. The results also provided basic and essential information about the contributing factors in this area. However, consideration of factors such as referral for treatment, home medications for pain relief, and outcomes of chronic pain are suggested in future longitudinal studies.

## Background

Chronic pain is defined as pain persisting or recurring for more than three months [1] in one or more than one anatomical region of the body, accompanied by emotional distress or serious functional disabilities and disrupting one’s everyday tasks and participation in social activities [2]. Chronic pain not only affects individuals’ daily activities, e.g. jobs, home lives, recreation, and communications [3], but has an impact on their quality of life and physical and mental well-being [4]. The negative consequences of pain are probably more significant in young adults, who are exposed to various stressors, life changes, and challenges in their future. According to a study, pain in young adults is associated with disability, low quality of life, and diminished efficiency [5]. In addition, pain-related issues for individuals, especially university students, often lead to drug abuse, opioid addiction, and mental disorders, e.g. depression [6].

Several studies have addressed the prevalence of pain and its correlation with health complaints in students and reported that pain is a common issue among university students which causes a decline in their psychological and social functioning and general well-being [7–9]. Studies conducted in the universities of Norway and Spain found that the prevalence of pain among the students was 54.7% and 30% respectively [10, 11]. The female students in Norway reported the highest incidence of pain (59.9%), and the students in Sweden were found to suffer from the highest rate of musculoskeletal signs of pain (63.8%) [10, 12]. Abledu and Offei (2015) studied 157 first-year nursing students at a school in Ghana and found the highest point prevalence of skeletomuscular pains to be in the subjects’ hands and wrists (15.3%) and backs (15.3%) [13]. In another study of 264 nursing students in Turkey (2017), 52.3% of the students complained of headaches, 42.4% complained of stomach pains, and 33% complained of back pains [14].

A study in Iran reported the rate of musculoskeletal pains among medical students to be 29.4% in the neck region, 24.3% in the upper part of the back, and 37.2% in the lower part of the back. The study also found a significant correlation between musculoskeletal pains and pains in the neck and knees based on the number of hours the subjects used their smartphones [15]. In another study, 39.4% of the medical students reported pain in their necks. Among the studied risk factors, such factors as age, carrying heavy bags, length of sleep, and number of hours spent using a computer had a significant correlation with pain in the neck and shoulders [16]. According to a study conducted in the east of Iran, failure to comply with the principles of ergonomics in designing seats for nursing schools resulted in great dissatisfaction with the seats and a musculoskeletal pain rate of 21.4% [17]. The results of studies of nurses in practice in hospitals in the northern and southern regions of Iran showed that the most prevalent musculoskeletal disorders are as follows: back pain (69–81%), neck pain (50-56.2%), and knee pain (48.9–63.5%). These chronic pains were related to various factors, including frequent bending, transferring patients, lifting and carrying medical equipment, bathing the patients, and lack of knowledge of the principles of ergonomics [18–20]. In a study by Choobineh et al., serious psychological needs, poor decision making skills, and lack of social support were more common among the nurses with chronic pains [18]. Arsalani et al. reported that poor control over their excessive workload and low job satisfaction led to increased work stress, emotional/mental/social issues, and increased risk of musculoskeletal disorders among nurses [21].

Research shows that medical and nursing students are more prone to chronic musculoskeletal pains than other students, which is the result of the nature of medical education. To acquire professional knowledge and skills, medical and nursing students must go through clinical training courses in the hospital environment. These courses are known as the most stressful part of medical education [22–24]. The stress of clinical training, long periods of standing next to patients’ beds, wrong twisting of the body during medical examinations or caregiving, and moving the patients [22, 24, 25], in addition to reading, writing, and using computers for long periods make this group of students more prone to pains in the back and neck than non-medical students [26, 27].

A variety of risk factors associated with chronic pain have been reported, among them demographic-social, psychological, clinical, and biological factors. Identification of these risk factors can help develop measures designed to prevent or manage the contributory factors in university students’ exposure to chronic pain [28].

One of the psychological risk factors is anxiety and depression, which was found to be more common among nursing students with chronic pains than those who did not suffer from these pains [29]. However, in some other studies, there was not a significant difference between the students with chronic pains and the students without chronic pains in terms of anxiety and depression [30, 31]. Other studies reported that the academic and psychosocial performance of students with chronic pains was less satisfactory compared to other students and the former experienced higher levels of anxiety and depression [32–34]. According to some other studies, students who suffer from chronic pains are more prone to absenteeism [29, 31].

Emotional intelligence may act as a protective factor against the negative outcomes of chronic pain [35]. Moreover, social support interventions as part of a multidisciplinary approach would be beneficial in coping with experiences of chronic pain [36]. However, a study by Feldman (2020) showed that, even though the students with chronic pains had less social support and support from their friends and other important people in their lives than the students who did not have chronic pains, the difference was not significant [37].

Yet, few studies have investigated influential factors in chronic pain among nursing students. There is need for more research into chronic pain in nursing students as the nature of practical training can aggravate the students’ chronic pain [38]. Although previous research has focused on the relationship between chronic pain with negative psychological characteristics, such as depression and anxiety, little is known about the relationship between chronic pain and positive psychological characteristics, such as emotional intelligence, academic satisfaction and social support in nursing students. One of the most important steps in pain management is identifying the contributory factors, and the physical and mental health of nursing students plays a key role in the future health of a country [39]. Accordingly, the present study was conducted to identify the predictors of chronic pain among nursing students.

## Materials and methods

### Study design

The present study is a descriptive, cross-sectional work of research conducted on nursing students at the universities of medical sciences of Iran, between February and November 2019.

### Setting

The setting of the study was all the nursing schools in Iran. The bachelor’s nursing program lasts four years, eight semesters, in Iran, during which period 130 credits are to be taken. From their second semester, nursing students can take courses which involve training in clinical environments. The master’s nursing program lasts two years, four semesters, during which period 32 credits are to be taken. From their first semester, postgraduate students undergo training in clinical environments.

### Participants and sampling method

The population of the study consisted of all the nursing students in the nursing schools of Iran. Based on the findings of previous research and the assumption that at least 20% of nursing students suffer from chronic pain [39], and using a sample size formula and a loss to follow-up rate of 20%, the size of the sample was set at 1719.

Sampling was carried out in several stages. At first, all the nursing schools in Iran were divided into five regions based on their geographical location: north, south, east, west, and center. Next, each region was considered as a category and the categories with a larger share of the students had more subjects selected from them. Subsequently, from each region, two provinces were randomly selected via two-stage cluster sampling and then three nursing schools were randomly selected from those two provinces. Students were selected from each school on a random basis: the researchers acquired a list of the students’ names from the education department of each school and selected the students who met the inclusion criteria using a table of random numbers created by SPSS.

The inclusion criteria were being a student at a nursing school, being an undergraduate student from semester 2 to semester 8 or a postgraduate student from semester 1 to semester 4, and not having been on academic probation. The students who were not willing to participate in the study, had been transferred from another school, had been absent or on a break at the time of sampling, had a serious mental or physiological disease at the time of the study, or had a history of use of psychoactive drugs or sedatives were excluded. Drug abuse and abuse of psychoactive drugs and the resulting physiological and psychological dependence on these drugs can affect an individual’s perception of pain and chronic pain behaviors. Also, mental disorders can reinforce pain signals and make the symptoms more severe, resulting in a significant delay in diagnosing pain disorders. Moreover, Individuals with a chronic disease, including multiple sclerosis, diabetes, and cancer, are prone to chronic pain and it is possible that use of pain relievers, self-care activities, and other pain management interventions affect their perception of pain. Accordingly, these nursing students were excluded [40, 41]. 

### Data collection

The questionnaires were collected as follows: after getting permission from the dean of each nursing school, the fourth author (AM) went to the education department of the schools and acquired a list of the names of all the undergraduate students in semester 2 to semester 8 and the postgraduate students in semester 1 to semester 4. Next, a sample of the students who met the inclusion criteria was selected using a table of random numbers created by SPSS. The selected students received an envelope containing a paper about the objectives of the study, an informed consent form, and the self-report questionnaires. In addition, the students were informed face-to- face about the objectives of the study and asked to manually complete the demographics survey and the questionnaires if they were willing to participate in the study when they were at school and submit them to the secretary at the education department.

All the questionnaires were handed out and collected manually by the fourth author (AM). The questionnaires were completed in the middle of a semester. The present study was a national study and had been approved by the Iranian biomedical ethics committee website and the researchers had obtained the necessary permits from the university. Moreover, before manual distribution of the questionnaires, the required permits were obtained from the dean of each nursing school.

To minimize bias and contamination of data, the co-researcher was instructed to follow a standard protocol at the time of data collection and random sampling. Moreover, to minimize social desirability bias, the respondents were informed about the objectives of the study and assured that the questionnaires would be completed anonymously and all information would remain confidential throughout the study.

### Data collection instruments

#### The demographic factors

The collected data consisted of the students’ age, gender, marital status, education level, parents’ education level, place of residence, and grade point average (GPA).

### The characteristics of chronic pain

In order to measure chronic pain, the researchers used a chronic pain questionnaire which consists of several parts. Initially, the nursing students were asked the following questions: Do you suffer from recurring or continuing pain? Have you been experiencing pain and discomfort for three months or more? Has the pain affected your life and daily activities? Affirmative responses to the previous screening questions showed that the participants suffered from chronic pain. In part two, the participants were asked to describe the frequency of their chronic pain by selecting one of the following choices: “constant and nonstop,” “once or a few times per day,” “once or a few times per week,” or “once or a few times per month.” The third part of the chronic pain questionnaire consisted of questions about the location of the participants’ pain: head, face, hands, feet, neck, shoulders, wrists, ears, back, abdomen, chest, eyes, arms, knees, and ankles. In the next part, the participants were asked to rate the intensity of their pain in the past two weeks on the Visual Analogue Scale (VAS) from 0 to 10. These items were developed according to the International Classification of Diseases, 11th Revision (ICD-11) criteria [1]. In a study by Shaygan et al. (2020) in Iran, the face validity and content validity of the items were assessed by 15 nursing, anesthesiology, and pain experts. All the items were found to be clear and comprehensible by 89% of the evaluators, and the impact score of the items was greater than 1.5. The CVI and CVR of the questionnaire were reported to be 0.87 and 0.82 respectively. The reliability of the items was tested using the test-retest method with a 2-week interval and the result was a Cronbach’s alpha of greater than 0.74 [35].

The visual analog scale (VAS) is an accurate and reliable tool for measuring the intensity of pain. Especially its vertical version was considered to be easier for the participants to understand and to be more effective for determining the intensity of pain [42]. A score of 1–3 indicates mild pain, 4–7 indicates moderate pain, and 8–10 indicates severe pain. The Test-retest reliability of VAS has been reported to be satisfactory (ICC = 0.71–0.99); its concurrent validity has been found to be moderate (0.71–0.78) as compared with the numeric pain rating scale (NPRS) [42]. According to a study by Rezvani et al. (2012) in Iran, VAS is sufficiently accurate and can be completed in a short time, making it a more appropriate tool for measuring chronic pain. The correlation coefficient between VAS and the short form of McGill Pain Questionnaire (SF-MPQ) was reported to be 0.86 [43].

#### Spielberger state-trait anxiety inventory [STAI]

Spielberger’s State-Trait Anxiety Inventory, commonly known as STAI, consists of separate self-assessment scales which measure state and trait anxiety. The sub-scale of state anxiety comprises of 20 statements which evaluate the respondent’s feelings “at the moment of responding.” The sub-scale of trait anxiety comprises of 20 statements which evaluate the respondent’s general and usual feelings. Each statement is scored on a 4-point Likert scale: (1) not at all, (2) somewhat, (3) moderately so, and (4) very much so. The anxiety-present items were scaled from 1 to 4. However, the anxiety-absent items were scaled in reverse from 4 to 1. A score of 4 indicates great anxiety, and 10 items from the state anxiety scale and 11 items from the trait anxiety scale are scored accordingly. As for scoring the other items, a high score indicates absence of anxiety, and this applies to 10 items related to state anxiety and nine items related to trait anxiety. To calculate a respondent’s score for each of the two subscales—some items are scored reversely—researchers add up the sums of the 20 items in each subscale. Thus, the score range of each subscale ranges from 20 to 80. Spielberger et al. (1983) reported the internal consistency of the scale to be 0.86–0.95; the test-retest reliability of the scale with a two-month interval was reported to be 0.65–0.75. The construct validity of the scale was found to be satisfactory [44]. Abdoli et al. (2020) measured the reliability and validity of the Persian version of Spielberger State-Trait Anxiety Inventory (STAI) using 492 students. As for the internal consistency of the scale, the Cronbach’s alphas of trait anxiety and state anxiety were found to equal 0.88 and 0.84 respectively. In evaluation of the construct validity of the scale by the convergent method, STAI was compared with Beck Anxiety Inventory and the Cronbach’s alphas of trait anxiety and state anxiety were found to equal 0.64 and 0.64 respectively [45]. In present study, the reliability of the scale was obtained 0.89.

### Patient Health Questionnaire-9 (PHQ-9)

The Patient Health Questionnaire consists of nine items, each of which addresses one of the symptoms of depression according to DSM criteria [Diagnostic and Statistical Manual of Mental Disorders]. PHQ-9 is one of the most reliable instruments for diagnosing depression in chronic diseases. The items are scored on a 3-point Likert scale, ranging from always [3] to never [0]. The total score range is between 0 and 27. A score of under 5 indicates absence of depression, 5 to 9 indicates slight depression, 10 to 14 indicates moderate depression, and 15 and above indicates severe depression in the respondent. In 2010, Zuithoff et al. tested the construct validity and reliability of PHQ-9 on 1338 patients. The internal consistency of the questionnaire was found to be ICC = 0.88, and its test-retest reliability equaled *r* = 0.94, confirming that the instrument is valid for detecting anxiety disorders [46]. Dadfar et al. (2017) tested the validity and reliability of the Persian version of PHQ-9 on 130 outpatients with a mental disorder. The total score of the Persian questionnaire ranged from 0 to 27. The internal consistency and test-retest reliability of the questionnaire were 0.88 and 0.79 respectively. Evaluation of the convergent validity of the questionnaire resulted in 0.7 with the brief version of Beck Depression Inventory-13 (BDI-13) and − 0.35 with the World Health Organization-five Well-Being Index (WHO5). Confirmatory factor analysis proved the good fit of the data: CFI=-0.94, TLF = 0.93, and RMSEA = 0.06 [47]. In present study, the reliability of the scale was obtained 0.84.

### Bar-on emotional quotient inventory

This inventory comprises of 90 items and 15 sub-scales which measure emotional intelligence. Responses to the items are arranged on a 5-point Likert scale and, thus, each item earns a score from 1 to 5 (“Completely agree” =5 and “Completely disagree” =1). Some of the items with negative content are scored reversely. The total score for each scale is the sum of the scores for the items in that scale, and the total test score equals the sum of the scores for all the 15 scales. Higher scores on the test indicate the respondent’s greater success in the scale in question or the entire test and vice versa. The total score range is between 90 and 450. The validity and reliability of the inventory were tested by Dawada et al. (2009) on 243 university students in the U.S. The internal consistency of this instrument equaled a Cronbach’s alpha of 0.96; the consistency of the items ranged from 0.67 to 0.93. Evaluation of divergent validity and convergent validity showed that the construct validity of the inventory was satisfactory. This instrument has a significant direct correlation with the positive emotions scale and a significant inverse correlation with the negative emotions scale [48].

In a study by Nejati and Meshkat (2018), the validity and reliability of the Persian version of the Bar-on Emotional Quotient Inventory were tested using 600 university students. The translated inventory consisted of 90 items and 15 subscales. The items were scored on a 5-point Likert scale: Completely disagree = 1, Disagree = 2, Not sure = 3, Agree = 4, and Completely agree = 5. The range of the scores was from − 90 to 450. Evaluation of the content validity resulted in a CVR of 0.86 and CVI of 0.87. The results of confirmatory factor analysis proved the good fit of the data, and the Cronbach’s alpha of the instrument was 0.94. The study also reported the following: GFI = 0.95, CFI = 0.917, and RMSEA = 0.02. The reliability of the inventory equaled 0.89 [49]. In present study, the reliability of the scale was obtained 0.86.

### Academic satisfaction scale

Developed by Atashkar et al. (2014) in Iran, the Academic Satisfaction Scale evaluates academic satisfaction in students of medical sciences. The questionnaire consists of 20 items which directly and indirectly address the student’s perception of his/her major, internal and external motives for selecting that major, and academic, professional, and financial prospects. The items are scored on a 5-point Likert scale (the minimum and maximum scores for each item are 1 and 5 respectively). The total score range is between 20 and 100. The content validity of the questionnaire was verified by five experts after revision of the items according to expert feedback. The content validity ratio(CVR) and content validity index (CVI) were 0.87 and 0.88 respectively. The reliability was found to equal a Cronbach’s alpha of 0.89 [50]. In the present study, the scale was distributed among 30 students, and then its reliability was calculated using Chronbach’s alpha, yielding a value of 0.84.

### Procidano and Heller social support scale

Developed by Procidano and Heller in 1983, the Social Support Scale consists of 20 items which are scored based on three responses: “Yes”, “No”, and “I do not know”. The total score range is between 0 and 20. A higher score indicates greater social support for the respondent. A Cronbach’s alpha of 0.9 verifies that the scale possesses excellent internal consistency. This instrument also possesses satisfactory concurrent validity and correlates with psychological distress and social efficiency scores [51].

In a study by Aghamirli et al. (2020), the validity and reliability of the Persian version of Procidano and Heller Social Support Scale were tested. The 20-item scale consists of five subscales: support (5 items), caring (4 items), assistance (4 items), information (4 items), and feedback (3 items). The items are scored on a 6-point Likert scale, from Completely disagree = 1 to Completely agree = 6. The range of the scores is from 20 to 120, with higher scores indicating more social support from the respondents’ point of view. The results of confirmatory factor analysis showed that the construct of the scale had a good fit to the data and all the goodness of fit indexes were confirmed. The Cronbach’s alphas of the subscales of the instrument were reported to range between 0.87 and 0.88 [52]. In present study, the reliability of the scale equaled 0.87.

### Data analysis

The categorical variables were described by number [n] and percentage (%) and the continuous variables were described by mean and standard deviation (SD). At first, a univariate logistic regression was done for each variable and then the variables with p-values of less than 0.2 were entered into the multiple logistic regression analysis. In multiple logistic analysis, changes in the significance level of some predicting variables are often detected. Therefore, those variables that had a p-value of smaller than 0.2 in univariate logistic regression are entered into multiple logistic analysis. In this manner, all the relevant and potential predictive variables are studied [53, 54]. To determine which variables should be considered in the multivariate model of the study, the researchers used the above-mentioned criterion. Also, multivariate logistic regression analysis was used to investigate the adjusted association of explanatory variables with chronic pain. Because the dependent variable, i.e. chronic pain, was dichotomous, binary logistic regression was applied.

For univariate and multiple logistic regression odds ratios (OR) with 95% confidence intervals (CI) with p-value were reported. The Hosmer-Lemeshow statistic and was applied to assess goodness-of-fit model. P-value less than 0.05 was considered statistically significant. Figures were drawn in GraphPad Prism 8.0. All statistical analyses were performed with IBM SPSS Statistics 26 software (IBM Corporation, Armonk, NY, USA).

## Results

### The study population’s demographic characteristics

A total of 1,719 students were studied. The average age of the participants was 22.4 ± 2.96 years and their grade point average was 16.03 ± 1.18. The majority of the participants were female (61.7%) and single (87%). All of the participants were university students at the time of the study, with the great majority being undergraduates (88.8%). None of the participants was a PhD candidate (Table 1).

**Table 1:** Demographic characteristics of the participants (n = 1,719)

Variables	N	%
Gender		
Male	658	38.3
Female	1061	61.7
Marital Status		
Single	1495	87
Married	224	13
Education level		
Bachelor’s degree	1527	88.8
MA	192	11.2
Grade Point Average		
Free of pain		
10–15	454	27
16–20	721	42.8
Chronic pain		
10–15	186	11
15–20	358	19.20
Father’s Education*		
Bachelor’s degree and higher	507	29.6
Diploma and associate degree	792	46.3
Some high school	347	20.3
Illiterate	66	3.9
Mother’s Education*		
Bachelor’s degree and higher	462	26.9
Diploma and associate degree	732	42.6
Some high school	401	23.3
Illiterate	117	6.8
Place residence		
Non-dormitory	720	41.9
Dormitory	999	58.1
Pain Status		
Free of pain	1175	68.4
Chronic pain	544	31.6
Frequency of pain		
Permanent	35	6.43
Daily	152	27.94
Weekly	244	44.86
Monthly	113	20.77

*Note* Sample size varies due to non-responses (*n = 1712, missing = 7)

**Fig. 1:**
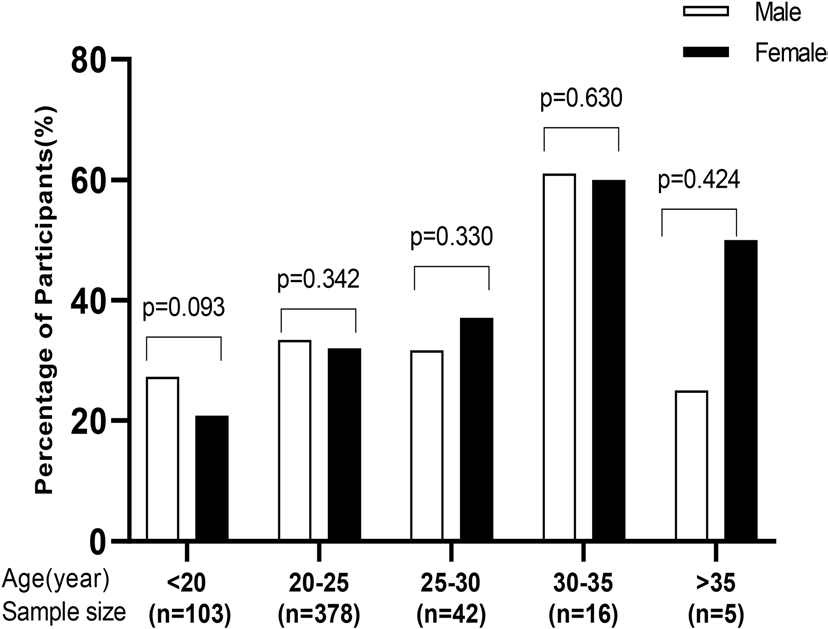
Comparison of gender and age group variables between the nursing students with chronic pain

The number of the nursing students who experienced chronic pain was higher in the 30–35 age group compared to the other age groups (*P* > 0.05), suggesting an increase in perceived chronic pain during adulthood. Moreover, there is not difference between male and female nursing students experienced more chronic pain in each age group (*P* > 0.05) ([Fig. 1).

### Prevalence and characteristics of chronic pain

**Fig. 2:**
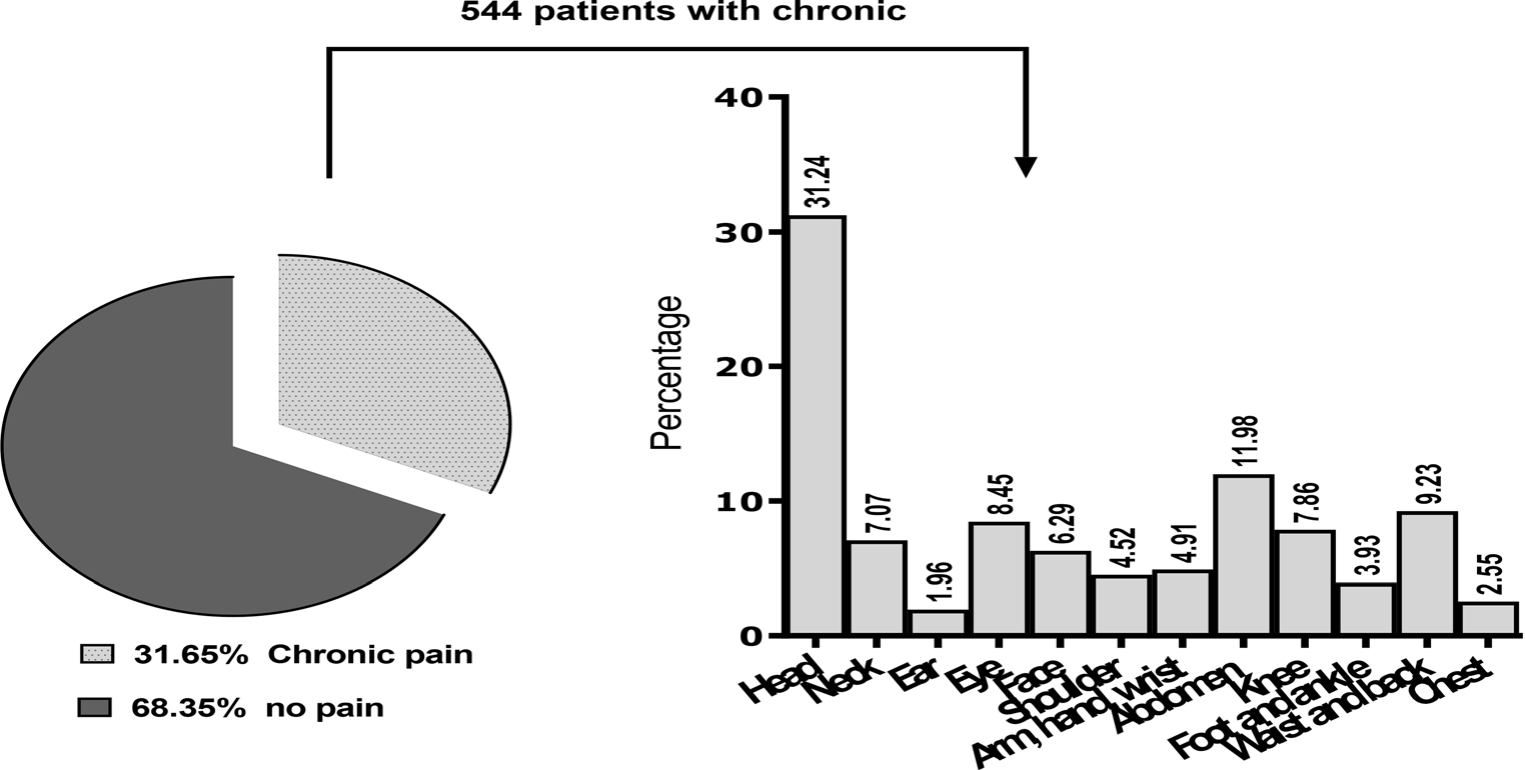
Self-expressed sites of chronic pain in nursing students

The results showed that 544 of the participants suffered from chronic pain, accounting for 31.65% of the cases (95% CI, 35–43). Severe chronic pain, defined as pain intensity of 8–10 on a scale of 0 to 10, was reported by 7.4% of the participants (95% CI, 0.063–0.087). Prevalence of chronic pain was higher among female (33%, 95% CI, 30–36) than male nursing students (30.1%, 95% CI, 27–34), but the difference was not significant (*P* = 0.23). Out of a total of 1,719 participants who were surveyed, 1175(68.4%) individuals were found to be free of pain. Among the individuals who had pain, 6.43% were suffering from permanent pain. Of the participants who had pain, 29.1% felt pain in a specific location. Most nursing students complained of chronic pain in the head [31.21] and abdomen [11.98]. Figure 2 shows the frequency of chronic pain regions as reported by the nursing students.

**Fig. 3:**
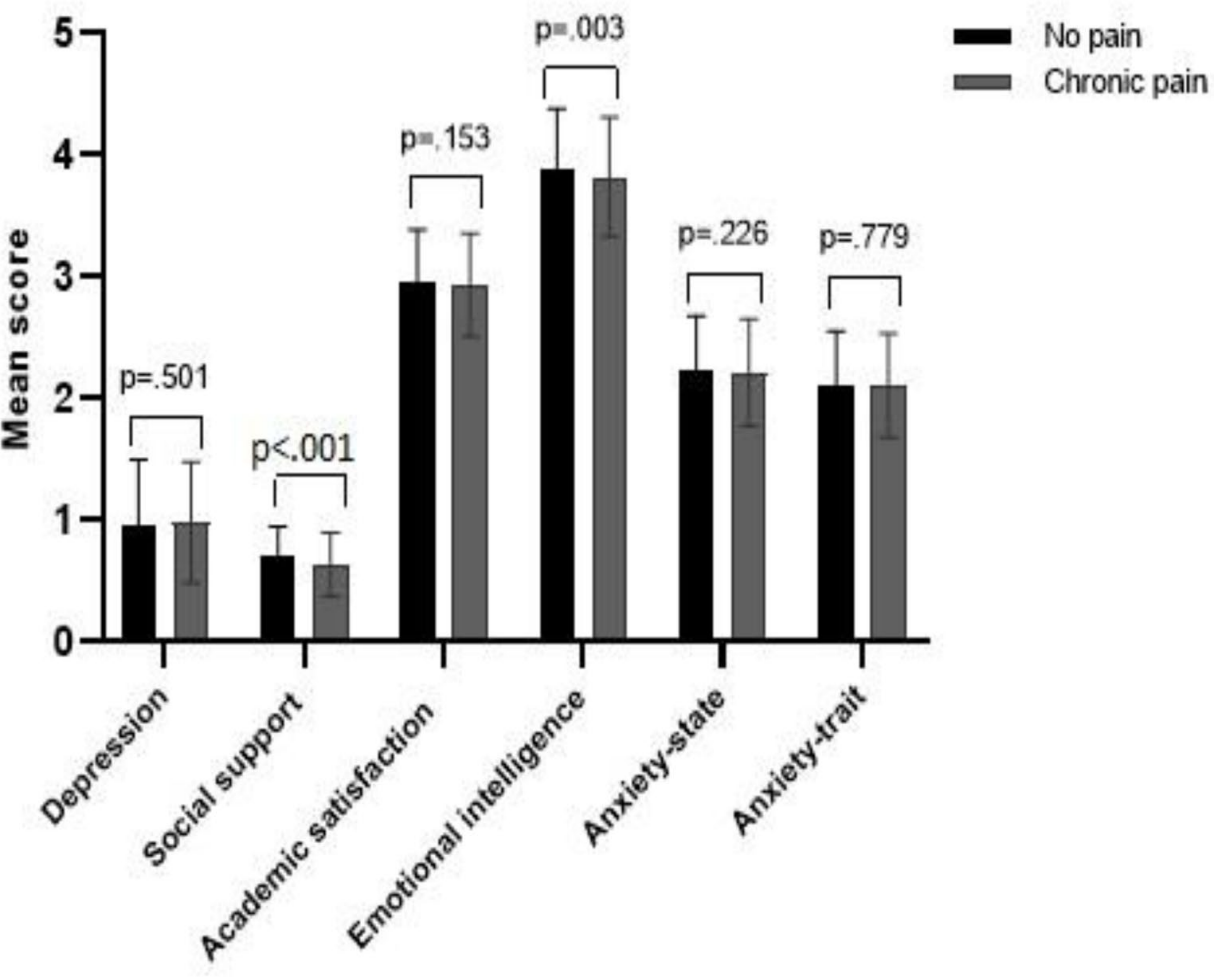
Comparison of educational-psycho-social variables between the nursing students with chronic pain and healthy ones

Comparing the mean of educational-psycho-social variables showed that the nursing students who had chronic pain were significantly different from the healthy ones in terms of social support (*P* < 0.001) and emotional intelligence (*P* = 0.003). However, there was no significant difference in mean scores of depression, satisfaction with education, state anxiety, and trait anxiety between the two groups (*P* > 0.05) (Fig. 3).

### Predictors of pain

In the univariate analysis, before assessing the predictors of chronic pain, the relationship between the demographic variables and educational-psycho-social factors, and the existence of chronic pain in nursing students were evaluated. The results showed that age, marital status, education level, social support, emotional intelligence, state anxiety, trait anxiety, and chronic pain were significantly associated in nursing students (Table 2). Then, at the third stage of analysis (multivariate analysis), binary logistic regression was performed. Between the three binary logistic regression models presented, the third model, with variables which included age, social support, state anxiety, and trait anxiety could predict chronic pain in the nursing students better than the other models (accuracy = 65.8%). The results of Hosmer-Lemeshow test (chi-square = 0.99 and *p* = 0.87) showed the multiple logistic regression models for this data was calibrated.

**Table 2:** Results of univariate and multiple logistic regression for predicting chronic pain in nursing students (n = 1,719)

Variables		Univariate logistic regression			Multiple logistic regression		
		β (SE)	*P* value	OR[95% CI]	β (SE)	*P* value	OR[95% CI]
**Age**		0.065(0.017)	**< 0.001**	1.07(1.03–1.12)	**0.064(0.024)**	**0.007**	**1.07(1.02–1.12)**
**Sex**	Female	0.071(0.107)	0.506	0.071(0.107)	-	-	-
	Male	-	-	-	-	-	-
**Marital status**	Single	0.432(0.147)	**0.003**	1.54(1.15–2.056)	0.275(0.171)	0.109	0.76(0.54–1.06)
	Married	-	-	-	-	-	-
**Education** **Level**	BD	-0.344(0.158)	**0.030**	0.71(0.52–0.97)	-0.177(0.215)	0.411	1.19(0.78–1.82)
	MA	-	-	-	-	-	-
**Mother’s Education**	BD and higher	-0.416(0.218)	0.056	0.66(0.43–1.10)	-	-	-
	Diploma	-0.225(0.208)	0.278	0.80(0.53–1.20)	-	-	-
	Some high school	-0.080(0.219)	0.278	0.92(0.60–1.42)	-	-	-
	Illiterate	-	-	-	-	-	
**Father’s Education**	BD and higher	0.067(0.289)	0.817	1.07(0.61–1.88)	-	-	-
	Diploma	0.149(0.281)	0.597	1.16(0.67–2.02)	-	-	-
	Some high school	0.243(0.295)	0.410	1.28(0.72–2.27)	-	-	-
	Illiterate	-	-	-	-	-	-
**Place residence**	Non-dormitory	0.173(0.110)	0.215	1.19(0.95–1.5)	-	-	-
	Dormitory	-	-	-	-	-	-
**Depression**		0.007(0.011)	0.501	1.01(0.99–1.03)	-	-	-
**Grade point average (GPA)**		0.030(0.044)	0.488	1.03(0.97–1.12)	-	-	-
**Social Support**		-0.50(0.010)	**< 0.0001**	0.95(0.93–0.97)	-0.049(0.011)	**< 0.0001**	**0.95(0.93–0.97**)
**Academic Satisfaction**		-0.010(0.007)	0.183	0.99(0.98–1.05)	-0.010(0.007)	0.173	0.99(0.98-1.00)
**Emotional Intelligence**		-0.011(0.004)	**0.003**	0.99(0.98–0.99)	-0.005(0.004)	0.274	0.98(0.97–1.11)
**State anxiety**		0.032(0.011)	**0.004**	1.03(1.01–1.05)	0.030(0.011)	**0.005**	**1.03(1.02–1.05)**
**Trait anxiety**		0.027(0.011)	**0.014**	0.97(0.95–0.99)	0.029(0.011)	**0.009**	**1.97(0.95–1.99)**

BD = Bachelor’s degree; OR = Odds Ratio; CI = Confidence Interval; SE = Standard Error; BD = Bachelor’s degree; Bold *P* values indicate significance at the 0.05 level

In this model, chronic pain was positively associated with age, social support, state anxiety, and trait anxiety (OR = 1.07, 95% CI: 1.02–1.12; OR = 0.95, 95% CI: 0.93–0.97; OR = 1.03, 95% CI: 1.02–1.05; and OR = 1.97, 95% CI: 0.95–1.99; respectively). Afterwards, the multinomial logistic regression test demonstrated that by including the aforementioned variables as predictors, the overall model fitted considerably better compared to an empty model (with no predictors) (*P* < 0.001). The results of this test revealed that despite the significant correlation of age, marital status, education level, and emotional intelligence with chronic pain in nursing students based on the chi-square test, when they were entered in the regression model, the impact of these factors was suppressed by the effect of age, social support, and anxiety (Table 2).

## Discussion

In the present, the majority of the nursing students were female and single, were studying for their bachelor’s degree, and lived in dormitories. The results showed that 31.6% of the nursing students suffered from chronic pain. The most common areas which were affected by pain were the head and abdomen. Kodana et al., report the prevalence of chronic pain in nursing students to be 79.2% [55]. . In a study by Abledu et al., 70.1% of the nursing students had suffered from musculoskeletal disorders in the past 12 months and 56.1% had been affected by the incapacitating consequences of pain. In addition, 44.6% of the students complained of pain in their necks, backs, lower backs, and wrists [13]. These differences in findings mainly resulted from methodological differences rather than population differences, including research design, data collection method, localization and definition of pain, different definitions of point prevalence, and different age groups. In addition, In Iran, clinical training courses for undergraduate nursing students start from their second semester. During their clinical training, nursing students should care for their patients, which entails standing for long periods. Also, at school, they have to spend long hours sitting while attending classes or studying. Poor posture during clinical practice and long hours of sitting in classes and libraries can account for the occurrence of musculoskeletal pain in nursing students [56]. Another reason for the differences between studies’ reports on the prevalence of chronic pain can be the different cultural and religious contexts of different societies. Religious beliefs can have a significant impact on individuals’ perceptions, emotions, and behaviors, as well as their health and sensitivity to pain [57]. Different cultural groups also differ in their manner of expressing pain. Some cultural groups may refrain from moaning, crying, or grimacing when they are in pain, while others openly manifest their discomfort in response to pain stimuli [58].

One study compared female undergraduate nursing and physiotherapy students’ beliefs about back pain in three different countries: Australia, Taiwan, and Singapore. It was found that fear of physical activates was different among the students of different countries. Compared to white Australians and physiotherapy students, Taiwanese and Singaporean nursing students had more negative beliefs about the consequences of back pain and were more afraid of physical activities [59]. These differences may be due to the collectivist cultural context, as opposed to the individualistic culture, of these populations and their different training programs. The focus of physical exercises on back pain management among physiotherapy students may reduce their fear of exercising and increase their positive adaptive behaviors regarding back pain [60].

In the present study, the prevalence of chronic pain among nursing students was less than in other studies. The students who were surveyed in the present study were from Iran, mostly lived in dormitories, and belonged to a collectivist culture. Collectivism is the dominant attitude in most Asian countries, meaning that individuals prefer to communicate their discomforts, including chronic pain, only in their families, are more tolerant of pain in the workplace or school, and satisfy their emotional needs only through their families to achieve a sense of unity, belonging, and identity. On the other hand, in European and North American countries, individualism is the dominant culture [61]. Cultural differences between collectivist and individualistic societies affect their rate of depression, emotional response to pain [43], and adjustment to stress and inconveniences, which can have an impact on individuals’ experience of pain, expression of pain, and response to pain [60]. As integral parts of a culture, religion and spirituality can facilitate adaptation to pain and reduce negative feelings, such as depression, which potentially aggravate pain [62]. Collectivist cultural values may positively correlate with such key psychological processes as self-adjustment, which can affect both the perception of musculoskeletal chronic pains and the associated disabilities [60]. It appears that collectivism can be a protective factor against chronic back pain and neck pain. Therefore, the authorities at universities should consider students’ cultural background in investigating and managing their chronic pain.

The results of the multinomial logistic regression test revealed that, when demographic characteristics and educational-psycho-social variables were entered in the regression model, there was a significant correlation between the variables of age, social support, and anxiety on the one hand and chronic pain in nursing students on the other. However, the students’ other demographic characteristics (marital status, parents’ education, GPA, and place of residence) and depression, academic satisfaction, and emotional intelligence did not significantly correlate with their chronic pain.

It was found that there was a significant correlation between chronic pain and age in the nursing students: older students were more prone to chronic pain. According to a study by Houde et al. (2016), there was a significant positive correlation between age on the one hand and pain and debility on the other in individuals with back pain. The correlation was stronger in the youth than in the elderly [63]. However, in their study, Duke et al. (2013) did not find a significant correlation between the university students’ age and the severity of their perceived pain [64]. According to a national study by Henderson et al. (2013) conducted in Australia, the prevalence of chronic pain in the 15 to 24, 25 to 44, and above 75-year-old age groups was 5%, 14%, and 36% respectively [65]. However, Boggero et al. (2015) reported a smaller prevalence of pain among the elderly [66]. In a study in Iran, the number of the adolescents who experienced pain was higher in the 12–15 age group compared to the 16–21 age group [67]. The discrepancy between the findings of the above-mentioned studies can be attributed to the studies’ use of different pain scales. Nonetheless, research shows that the rate of chronic pain is increasing across different populations and age groups [68].

Another finding of the present study was the presence of a significant correlation between social support and chronic pain in the nursing students. Even though the correlation was not very strong, the students who received more social support reported less chronic pain. According to a study by Zoghipaidar et al. (2020), perceived social support played a significant role in predicting the rate of chronic muscular pains in married women [69]. In their study of the impact of family support on the extent of pain and depression in 2,411 patients with arthritis rheumatoid, Hung et al. (2017) found that the patients who enjoyed the support of their family members and spouses showed significantly fewer symptoms of depression ad pain [70]. According to another study, the individuals who live close to their families or belong to a large family are more competent in controlling and managing their pain [71]. These findings show that social support, including the support of one’s family, friends, and other people who are important in one’s life, is a potential source of energy for coping with one’s chronic pain. Therefore, the role of social support in managing chronic pain should be underscored.

The results of the present study showed that there was a significant correlation between chronic pain and anxiety in the nursing students. Even though the correlation between the variables of state anxiety and trait anxiety on the one hand and chronic pain on the other was not very strong, state anxiety was found to increase the risk of chronic pain in the nursing students more than the variables of depression, social support, academic satisfaction, and emotional intelligence did. In a study by Makovec (2015), there was a significant positive correlation between back pain and anxiety in the participants: the individuals who reported moderate anxiety suffered from more pain [72]. Several studies have reported that anxiety can reduce the quality of life of patients with chronic pain [69, 73, 74]. Moreover, most individuals with chronic pain have at least one anxiety-related disorder [69]. As anxiety is associated with an increase in chemical transmitters, especially noradrenaline and adrenaline, which cause tension and muscle spasm, it can contribute to pain [75]. Accordingly, educational interventions designed to help nursing students manage their anxiety can decrease the rate of pain in this population.

In the present study, the variable of depression did not correlate with chronic pain in the nursing students. However, previous research showed that the prevalence of depression was higher in patients with chronic back pain than in the general population [76]. In their study of the relationship between anxiety and depression and perception of pain in women after mastectomy, Hansdorfer-Korzon et al. (2016) found that such mental factors as depression, anxiety, and belief system played a significant part in the patients’ perceived severity of pain [77]. These findings were confirmed by another study [78]. Sometimes, depression in individuals with chronic pain remains undiagnosed and is, therefore, not treated [79]. In view of the inter-correlation between chronic pain on the one hand and anxiety and depression on the other, the significance of screening individuals with chronic pain for mental health problems should be underscored [80]. Differences between the screening instruments for depression employed in the above-mentioned studies and the present study may account for the discrepancy between the findings.

In the present study, it was also found that emotional intelligence did not significantly correlate with chronic pain in the nursing students. However, Wright and Schutte’s 2014 study of the moderating effect of emotional intelligence and self-efficacy in patients with arthritis who were visiting a pain a clinic in Australia because they were suffering from chronic pain showed that emotional intelligence and self-efficacy correlated with better pain management and reduced mental perception of pain [81]. The results of a study by Kopera et al. (2018) showed that, among the alcoholics who visited a rehab center in Poland, those who were better at moderating their emotions and had higher education experienced less pain [82]. According to another study (2020), emotional intelligence, self-confidence, and parental models were influential factors in the development and persistence of chronic pain in adolescents [35]. By reducing emotionality or negative moods, higher levels of emotional intelligence can decrease pain vulnerability or perception of pain [83]. It may emphasize the need for strategies to increase emotional intelligence in nursing students. Additional research is required to determine whether training in emotional intelligence could provide direct symptom relief or even potentially serve as a protective factor, reducing pain vulnerability in nursing students who may have other identified risk factors for the development of chronic pain.

One of the strengths of the present study was its large sample size, which confirmed the findings related to chronic pain changes. Another important strength of the study was that, in addition to assessing the prevalence of chronic pain in the nursing students, the educational-psycho-social factors contributing to the incidence of chronic pain were evaluated.

One of the limitations of the present study was personal differences between the nursing students, including differences in their physical, and family conditions, which may have affected the results of the study. In addition, lack of research on chronic pain and factors associated with it in the present study population restricted the possibility of comparing the findings of the present study with similar studies. Additionally, some factors such as sitting time, sitting straight, academic semester, and using assisting devices during reading in university, were not taken into account in the present study. Other limitations of the study were that completing the questionnaires was time consuming and the questionnaires were self-report instruments, in which participants may report their pain to be higher or lower than the real levels. Therefore, before distributing the questionnaires, the researchers informed the students about the objectives and purposes of the study and asked them to complete the questionnaires at their convenience.

## Conclusion

The findings of the present study showed that social support can lessen nursing students’ vulnerability to chronic pain. On the other hand, increasing age and experiencing anxiety during their education increase the risk of chronic pain among nursing students. The findings of the study corroborate some evidence of the impact of age, psychological factors, and social support on chronic pain among nursing students. Thus, for effective prevention of the risk of developing chronic pain by nursing students, the role of social support and education-related anxiety must be taken into account. The findings of the present study can help university authorities take the necessary steps to manage chronic pain in students who suffer from pain and take preventive measures for students who are free from pain, which will contribute to the students’ academic performance and success. It is suggested that future research should use cohorts in order to identify the effective factors in pain and appropriate interventions for relieving nursing students’ chronic pain in other societies to add to the existing knowledge.

## Data Availability

The dataset generated and/or analysed during the current study are not publicly available due to promises of participant anonymity and confidentiality but are available from the corresponding author on reasonable request.

## Abbreviations

GPA: Grade point average
VAS: Visual analog scale
NPRS: The numeric pain rating scale
PHQ-9: Patient Health Questionnaire-9
OR: Odds ratio
CI: Confidence interval
